# Shifting the Paradigm: The Dress-COV Telegram Bot as a Tool for Participatory Medicine

**DOI:** 10.3390/ijerph17238786

**Published:** 2020-11-26

**Authors:** Michela Franchini, Stefania Pieroni, Nicola Martini, Andrea Ripoli, Dante Chiappino, Francesca Denoth, Michael Norman Liebman, Sabrina Molinaro, Daniele Della Latta

**Affiliations:** 1 Data Learn Lab, Institute of Clinical Physiology of the National Research Council, 56124 Pisa, Italy; michela.franchini@ifc.cnr.it (M.F.); francesca.denoth@ifc.cnr.it (F.D.); sabrina.molinaro@ifc.cnr.it (S.M.); 2 Data Learn Lab, Gabriele Monasterio Foundation, 1, 56124 Pisa, Italy; nicola.martini@ftgm.it (N.M.); ripoli@ftgm.it (A.R.); radio1@ftgm.it (D.C.); dellalatta@ftgm.it (D.D.L.); 3 IPQ Analytics, Kennett Square, PA 19348, USA; michael.liebman@ipqanalytics.com

**Keywords:** COVID-19, SARS-CoV-2, participatory medicine, co-morbidity profile, telegram bot

## Abstract

Severe acute respiratory syndrome coronavirus 2 (SARS-CoV-2) pandemic management is limited by great uncertainty, for both health systems and citizens. Facing this information gap requires a paradigm shift from traditional approaches to healthcare to the participatory model of improving health. This work describes the design and function of the Doing Risk sElf-assessment and Social health Support for COVID (Dress-COV) system. It aims to establish a lasting link between the user and the tool; thus, enabling modeling of the data to assess individual risk of infection, or developing complications, to improve the individual’s self-empowerment. The system uses bot technology of the Telegram application. The risk assessment includes the collection of user responses and the modeling of data by machine learning models, with increasing appropriateness based on the number of users who join the system. The main results reflect: (a) the individual’s compliance with the tool; (b) the security and versatility of the architecture; (c) support and promotion of self-management of behavior to accommodate surveillance system delays; (d) the potential to support territorial health providers, e.g., the daily efforts of general practitioners (during this pandemic, as well as in their routine practices). These results are unique to Dress-COV and distinguish our system from classical surveillance applications.

## 1. Background

December 2019 saw the appearance of a novel coronavirus, coronavirus disease 2019 (COVID-19), caused by severe acute respiratory syndrome coronavirus 2 (SARS-CoV-2). It emerged in China and quickly spread globally. COVID-19 was declared a pandemic by the World Health Organization (WHO). As of 31 August 2020, there were over 25 million confirmed cases globally [1]. The initial spread of the virus in Italy was noted on 22 February, even though some atypical pneumonia cases had been detected earlier. The number of related cases continued to increase until the Italian government imposed lockdown measures on 9 March. Given the relevant proportion of infected people needing hospitalization in intensive care units (ICU), COVID-19 is a crucial issue impacting the national healthcare system’s capacity. Because of this, Italy formulated proportioned and controlled measures to guarantee adequate funding to increase the number of ICU beds and the production of personal protective equipment [2].

SARS-CoV-2 pandemic management is limited by great uncertainty, for both health systems and citizens. The number of infected people is currently well known, when referring to those who require hospitalization, but the true number of infected citizens, including asymptomatic and pauci-symptomatic individuals, who may not have required hospitalization, remains unclear. 

One of the current challenges for the public health service is to identify the most effective methodology for detecting the total number of COVID-19 cases, by (a) integrating all available information sources and identifying others that could prove valuable, and (b) following the 3T approach (tracking, tracing and treating), which proved to be a winning strategy during the West African Ebola outbreak [3].

Public health modeling of the spread of COVID-19 has shown that successful contact tracing can reduce the number of infections dramatically, if infected individuals are quarantined, and their contacts and places visited can be identified. Despite the importance of contact tracing in controlling the spread of COVID-19, some limitations may hamper its success [4]. First, many infected individuals are not identified and contact tracing might be delayed in capturing enough new infections to slow transmission. Infections could develop in individuals, not identified as contacts, and continue to seed new hotspots. Second, contact tracing is operationally difficult and costly to scale to critical levels needed when the situation yields many contacts, and where they are spread across disparate locations. As a result, each newly infected individual yields several additional contacts, leading to hundreds of exposures. Consequently, as the pandemic spreads, the number of contacts can increase exponentially. The scaling of contact tracing to keep pace with this growth can be very difficult, with a greater likelihood that critical contacts will be missed or lost to follow-up [5]. 

Presently, in many states, the local Department of Health (DoH) collaborates with community partners to increase the capacity for additional calls to bolster the limited pool of full-time employees available to trace the contacts of infected individuals. 

Several countries have introduced mobile-phone based apps and digital platforms to aid surveillance activities for COVID-19 control. Examples are Immuni in Italy, Alipay Health Code in China, Social Monitoring in Russia, Corona 100m in South Korea, and Trace Together in Singapore—all of which have been developed to perform complementary functions: syndromic surveillance and contact tracing [6]. The Immuni app is driven by the national surveillance system, i.e., when local health authorities register a new coronavirus case, they enter a code into the system, with the consent of the patient. The system then automatically sends notifications to users who have been in close contact with the positive individual.

Expanding the workforce and strategies that support contact tracing is a good solution, but individual concerns about contact tracing and related issues, e.g., privacy and data security, could still critically impact the success of contact tracing programs and, thus, the potential for containment of the pandemic [4].

To address this challenge, we incorporated participatory surveillance that has shown promising results in several public health events. The use of a collaborative information pathway has provided a rapid way for the data collection on symptomatic or paucisymptomatic individuals to complement traditional health surveillance systems [7,8]. According to the participatory medicine theory, defined by Gilles Frydman and colleagues [9] as “a movement in which networked patients shift from being mere passengers to responsible drivers of their health, and in which providers encourage and value them as full partners”, participation could improve a proactive approach to health through a consensus-building method, based on the notion of “power with” for fostering more empowered communities [10]. Empowerment, toward this goal, is not considered as the external and altruistic transfer of power in the name of the common good (conservative health promotion approaches), but it is the result of simultaneous changes at individual and population levels aimed at social transformations, and aligned with a critical Health Promotion approach [10].

Health promotion, as a conceptual framework, refers to any disease population being divided into four groups (a) healthy population, (b) population with risk factors, (c) population with symptoms, and (d) population with disease or disorder, and targets each group with specific interventions to comprehensively address the need of the whole population [11]. In any group, health promotion is supported by health education, the practice of providing the most correct information and knowledge to individuals and communities, and providing skills to enable individuals to adopt healthy behaviors voluntarily [11].

Currently, the combined technology offering and its widespread access (smartphone, tablet, PC) across all segments of the population establishes a great opportunity to implement an innovative system for assessing socio-health data, promote participation, and support health education. “Population health technology” is a recent umbrella term subsuming diverse technologies that have the potential to improve early (and big) data gathering to enable detection of early patterns indicative of potential disease outbreaks [12]. Examples of tools developed in support of participatory medicine have been available for many years, e.g., the web-based platform CureTogether, founded in 2008. On this platform, users quantify and share information about the nature and severity of symptoms, as well as responses to treatment. The site then aggregates and analyses (anonymized) data so that users can see which treatments work for people with similar symptoms, co-morbidities, or demographics [13], in alignment with the Predictive, Preventive, Personalized and Participatory (P4) medicine framework [14,15].

With the worsening of the COVID-19 pandemic, in spite of interventions to control the spread of infections, e.g., testing and social isolation, we are faced with an underestimation of cases, delay in case notifications, and public concerns about privacy and data security. Alternative sources of up-to-date information could provide a complementary solution for assessing the behavior of communities towards the pandemic, establishing the risk profile of contagion, understanding the progression of the disease, spreading educational messages, and supporting community-based interventions [6].

Our idea for innovation facing the COVID-19 pandemic aligns with the concepts of participatory surveillance, health education and promotion, and community empowerment. This bottom-up approach enables information to be directly acquired with the collaboration of the citizen (in relation to their daily habits), associated with COVID-19 risk. It also considers other personal risk factors, including the occupational and psychological domains.

Moreover, the participatory approach implies that evaluating the data provided by the users allows the system to quickly return useful information, to safeguard their individual health, and the health of their community, within a cooperative framework.

The Doing Risk sElf-assessment and Social health Support for COVID (Dress-COV) system, based on the Telegram bot, was designed and developed to understand, catalogue, and scientifically assess parameters potentially related to the disease process, extending from risk assessment for SARS-CoV-2 infection to the actual disease and outcomes of COVID-19 [16]. Dress-COV was mainly developed as a tool to model the complexity that exists, but may not be apparent in cross-temporal analysis along the dynamic process of COVID-19. Characterization of positive symptomatic or pauci/asymptomatic case progression and correlative analysis of individual co-morbidities and social habits are critical to establishing risk profiles related to the SARS-CoV-2 infection and the most severe disease complications [17]. Identifying such risk profiles should support the establishment of protective strategies against the infection for those not impacted, but who share the same risk characteristics.

Dress-COV not only alerts users to their own risk level, but also suggests potential behavior modifications that can lower that risk, based on critical, scientifically validated information that is constantly updated. The system was designed to establish a lasting link between the user and the tool, aimed at directing the citizen’s conduct towards protective strategies for their own general health and with particular containment of the risk of SARS-CoV-2 infection, through a self-empowered management of personal actions, in case of quarantine notification delay by the local DoH. The Dress-COV system aims to stimulate the process of self-empowerment by allowing a genuine shift of power from traditional experts to the citizens, through a process of a consensus-building method, aimed at cooperative searches for information and solutions that meet both parties’ interests and needs [10,18]. This process assumes that users trust the privacy and data security measures adopted by the tool, and it would be facilitated by efficient methods to administer the survey, which can be quickly adapted to new information needs. This tool incorporates and relies on an intelligent interface and specific functions that can be rapidly enhanced and implemented based on the suggestions and interests of users. Dress-COV system meets these requirements.

This paper describes: (1) the Dress-COV system design and features; (2) its current status and initial results, although currently reflecting a limited number of registered users, and reports its potential for collecting and sharing information; (3) its potential for promoting community knowledge, empowerment, and informing public policies; (4) its planned evolution and potential role in enhancing the participatory aspects of public health policy; (5) its current system limitations and envisaged solutions to overcome them.

## 2. Materials and Methods

### 2.1. System Design and Functions

The Dress-COV system design was based on a user-centered approach, using a goal-oriented design (GOD) methodology [19], in which end users and stakeholders guide the process, as they will ultimately validate the final product. Compared with task-oriented systems, goal-oriented systems more strictly reflect human motivations rather than technology; goals are not expected to change significantly over time, whereas tasks are constantly changing. The design process involved six different phases, summarized below:

(1) Identification of system technical features: the Dress-COV system was developed using the Telegram cloud-based messaging application that securely routes all communications through encrypted servers in the cloud.

Specifically, it was developed using the bot technology offered by the Telegram messaging application. It is possible to create a virtual user who can exchange messages with other users using the Telegram bot Application Program Interface (API) and develop third-party software with Artificial Intelligence (AI) features. Virtual users access the network and communicate through the same channels available to the users. They are able to perform actions in an automatic way, can collect data, provide answers to user queries, inform people making decisions, and assess risk behaviors in different scenarios by querying users.

**Figure 1:**
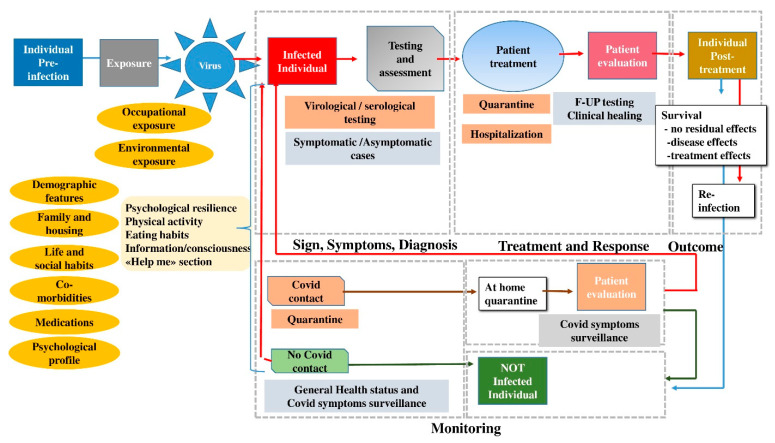
Doing Risk sElf-assessment and Social health Support for COVID (Dress-COV) conceptual model.

(2) Identification of the conceptual reference model: the Dress-COV system has been developed following a logical scheme, placed in a cross-temporal manner along the dynamic process of COVID-19. This conceptual reference model aims to characterize the Dress-COV user in the various stages of the disease process, from the risk assessment to the disease outcome (hospital discharge, healing, reinfection, etc.), also providing information for the monitoring of uninfected individuals (Figure 1). Currently, data about treatments for COVID-19 are not incorporated within Dress-COV, such as prediction of potentially fatal outcome. We plan to enhance the Dress-COV database by integrating other information resources provided by the Regional Health System and/or general practitioner (GP) data sources. This planned and ongoing upgrade of the system will lead to further releases that will address chronic care.

In particular, the integration with other sources of data allows for better segmentation of Dress-COV users into distinguishable groups of risk based on (a) health status using those relevant and validated data derived from the user’s contacts with the Italian National Healthcare Service (NHS), before the COVID-19 onset, (b) health and social information derived from GP medical records. The integration of the Dress-COV database with other sources of data will be performed anonymously, by using a series of key IDs for users providing their consent (to being linked to their GP health records). This allows users to earlier report potential COVID-19 symptoms, along with their risk profiles, and help the GPs to evaluate the user’s health status following a patient-centered, proactive approach.

**Figure 2:**
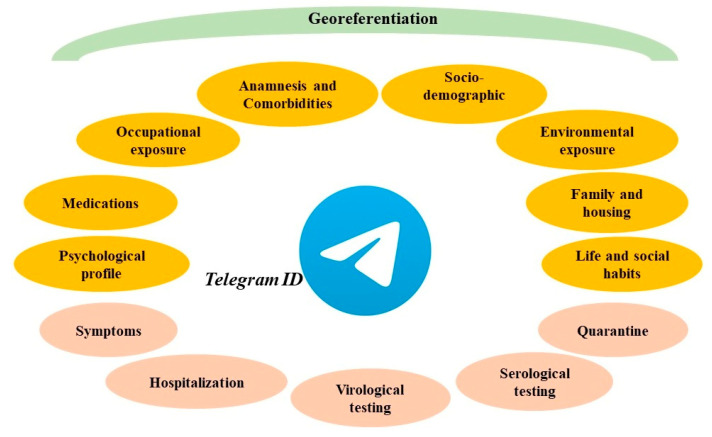
Dress-COV survey sections.

(3) Design and test of the epidemiological survey: Dress-COV collects data by asking users a set of more than 200 questions, grouped into 30 clusters of 7 daily questions. This is an innovative and effort-saving method to administer the questionnaire via bot over several days, and which increases the consciousness about each proposed question and user compliance. For each question, the bot presents the several response options. These can be activated with a simple click, without requiring users to submit text. There are also some control questions to assess the degree of reliability of the responses. As shown in Figure 2, Dress-COV questions include all sources of exposure that could lead to the infection by SARS-CoV-2 and all the personal aspects that could promote the onset of severe complications of COVID-19. Moreover, Dress-COV aims to evaluate the diagnostic pathway (virological and serological testing, quarantine) followed by the user and the possible occurrence of hospitalization for COVID-19 complications. Questions also concern those COVID-19 symptoms currently known. The system capability of modifying the original structure of the proposed items allows for updating the tasks dedicated to the symptoms collection, according to the new scientific evidence that will emerge. A detailed description of the survey items is provided in Supplementary Materials. 

According to the goal-oriented design methodology, the Dress-COV survey has been initially tested on a sample of 100 users before launching the system into the live environment.

**Figure 3:**
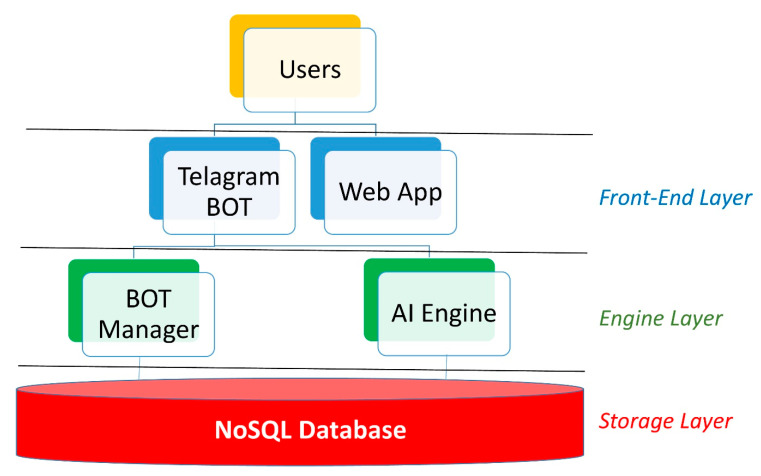
IT architecture overview.

(4) Definition of the user needs and focus: in order to improve the citizens’ participation and to shore up the long-lasting link between the user and the tool, the Dress-COV not only investigates the users’ requirements for being supportive of their needs (e.g., shopping, housecleaning, drug supplying), but also suggests solutions for addressing those needs.

(5) Definition of the IT architecture: the IT architecture is composed of three layers: the front-end, the engine, and the storage layer, as shown in Figure 3.

The storage layer uses MongoDB, one of the most widely adopted NoSQL databases. NoSQL databases have become wildly popular among modern application development teams. Typically, open source and built for cloud, and distributed computing, this generation of databases allows for agile development, supports dynamic unstructured data, and horizontal scaling. In particular, MongoDB is a collection-oriented, schema-free document database. Data are grouped into sets termed “collections”. Each collection has a unique name in the database, and can contain an unlimited number of documents. The database consists of four separate collections: registered users, questions submitted to the users, responses coming from the users, and the mapping of Italian regions and provinces. The question collection stores for every question (a) the question body, (b) the response options, (c) some attributes that support the logic used to present these questions, i.e., the association of the question to the block that groups it together with others in the presentation to the user, and the position of the question itself within the block. Moreover, the question collection maintains attributes that specify the precedence logic according to the responses already provided by the user.

The management of bot automation, the database incorporation of third-party sources, and the analysis of the data stored in the database, take place within the engine layer. In detail, a series of algorithms, in Python, have been developed to enable the user to communicate with the database through a Telegram bot and integrate the aforementioned with COVID-19 data coming from both literature and institutional sources. At the same time, a series of Python algorithms designed to train AI based models, both supervised and unsupervised, have been implemented to analyze the acquired data and be enabled to propose new predictive risk models.

The data request and the information transmission take place within the front-end layer. The Telegram bot APIs allow communication with the users who join the Dress-COV system. Thanks to a user profiling process, it is possible to define the administration users and allow them to analyze the data in real time through special web apps, which could play an important role in the dynamic definition of the survey and in the planning of regional interventions.

(6) Definition of the risk assessment process: this is divided into three phases (a) Dress-COV accumulates experience by integrating the responses provided directly by the users; (b) Dress-COV, based on the accumulated experience, progressively improves the ability to assess the level of individual risk, and the proposed questions become increasingly detailed enabling the estimation of personalized risk; (c) Dress-COV becomes a real expert system. The artificial intelligence models that produce this analysis are fully trained, with increasing validation, based on the number of users who join the system.

### 2.2. Recruitment

To facilitate participant recruitment to Dress-COV, local municipal leaders, community leaders, general practitioners, and public health services have been involved. Furthermore, various communication channels have been used to promote voluntary participation among those people with either an Android or IOS smartphone. The tool was first introduced to the general population on 9 May 2020, through a press conference. It was subsequently disseminated using the main social channels. Currently, the dissemination phase continues.

Ethical Statement: According to the General Data Protection Regulation, in Italy, the management of sensitive personal data referred to identify individuals is legally authorized only by those organizations belonging to the NHCS. The use of personal data for scientific purposes is only permitted in anonymous form after the positive evaluation of an ethics committee. The Dress-COV has undergone successful evaluation by the CEAVNO ethic committee of Pisa (protocol n. 17714 on 9 July, 2020).

### 2.3. Statistical Analysis

Referring to the phases (b) and (c) of the risk assessment process, we will apply and validate machine learning (ML) models that accurately estimates two different risk scores: (1) the risk of infection by SARS-CoV-2, and (2) the risk of severe symptoms by COVID-19. Traditional ML algorithms, such as the Random Forest (RF), the Support Vector Machine (SVM), and the Gradient Boosting Machine (GBM), will be trained in a supervised manner to make predictions based on the learned patterns from labeled data [20].

Towards this goal, the Dress-COV database will be parsed into two sets, i.e., a percentage of the dataset available for training the algorithm, and a testing set, i.e., remaining part of the dataset on which to test performance and evaluate the results in terms of accuracy.

To test the effectiveness of this technique for verifying the representativeness of our train data versus our test group, we will use the area under the receiver operating characteristic curve (AUC-ROC) for describing how effective the model is in distinguishing individual features among people with positive and negative virological and serological testing. Feature importance analysis will be performed to examine which of the input variables had the most predictive power.

Due to the current limited number of users of the Dress-COV system, we are performing the training and testing of ML algorithms by including a curated external dataset of 2703 respondents coming from the EPICOVID19 survey [21,22]. The EPICOVID19 dataset is composed of several variables with the same meaning of the Dress-COV ones, so we apply a coding procedure in order to standardize data dictionaries for making user responses comparable between the two datasets. The indication of the common variables is provided in Supplementary Materials. The obtained dataset will be used to identify, among the common variables, the most predictive of the risk of infections and/or severe symptoms. The predictive value of the variables collected by Dress-COV only will be evaluated once the number of users increase.

Referring to phase (a) of the risk assessment process, we performed descriptive statistical analysis concerning both process and outcome indicators. Results are shown in terms of percentages that reflect the 95% CI, calculated using the Clopper–Pearson (exact) method. Data of the users who will not complete the process of data collection will be excluded from all the phases of the risk assessment process. 

## 3. Results 

### 3.1. Recruitment

**Figure 4:**
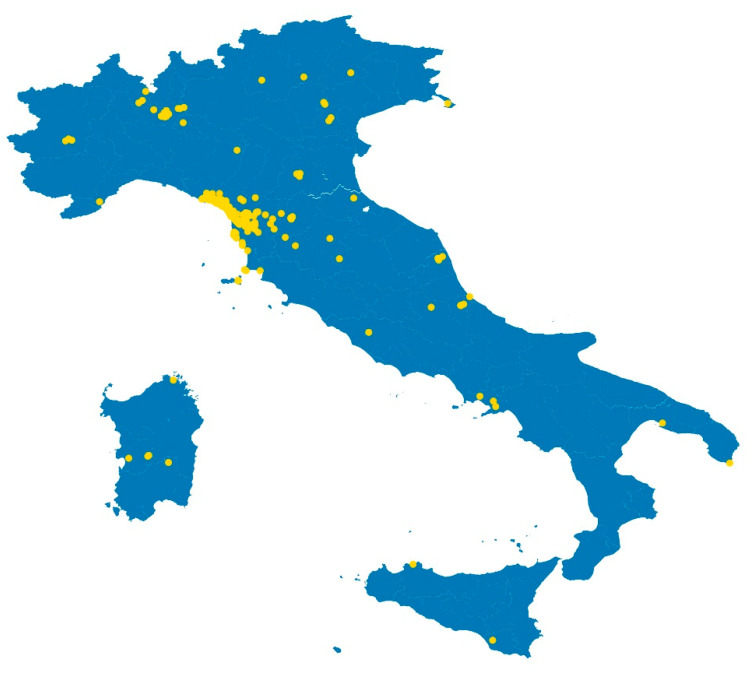
Dress-COV: users’ distribution in Italy.

The number of users was 489 on 27 August: 389 people had already completed the previous survey question. The users were primarily male (54.0%), and primarily between 40 and 59 years of age (51.0%). Compliance to the tool was 99.8%, essentially all of those who accessed Dress-COV completed the survey and submitted their information. The geographical distribution of the Dress-COV Italian users is shown in Figure 4.

### 3.2. System Functions

In coherence with the system design, a set of functions, of interest to the users, is being made available.

**Figure 5:**
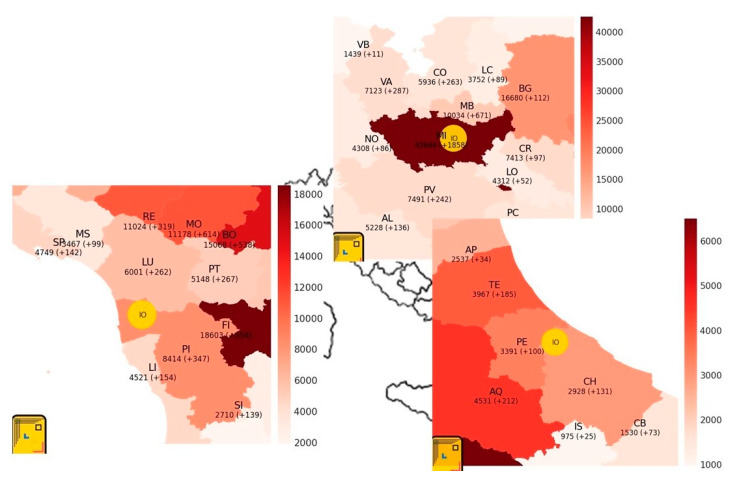
Dress-COV: daily visualization of contagions, in terms of total numbers and daily increase. Example of views from different regions.

During usage time, Dress-COV provides users with some relevant information regarding the number of infections by geographical area (as supplied by the national health surveillance system). By default, the user visualizes the daily situation of infections based on the physical location they declared when first accessing the system. Users can upgrade their position (in Italian “aggiorna la mia posizione”) at any moment, and can visualize the infection situation of other geographical areas (Figure 5) (in Italian “pianifica uno spostamento”).

**Figure 6:**
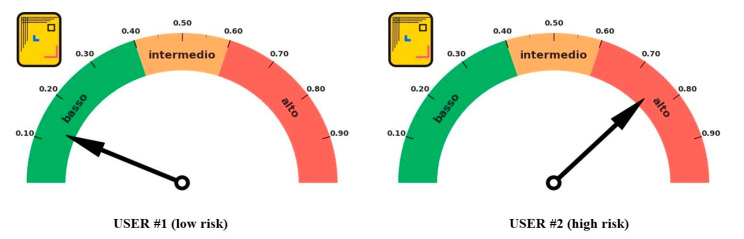
Dress-COV: examples of individual reports about the risk of being a Covid-19 case.

Although the AI based risk assessment process development (phases b and c of the risk assessment process) is still under development, since 25 May 2020, Dress-COV has sent users a risk estimation for becoming a COVID-19 case (Figure 6). The risk estimation is based on the algorithm previously developed by Menni and colleagues [23]. Users, at their discretion, can receive their individual risk indicator by activating the specifically developed function of the bot (in Italian “calcola il mio rischio di contagio”).

Finally, the Dress-COV system functions allow users to delete all of their own data in accordance with the General Data Protection Regulation, as well as communicate with the Dress-COV team by sending feedback, suggestions, and asking questions.

### 3.3. Descriptive Statistical Analysis

Referring to the phase (a) of the risk assessment process (Dress-COV aggregates and learns from its use by integrating the responses provided directly by the users), some descriptive analysis is performed in real time and users receive periodic updates based on data extracted from the Dress-COV respondents (age, co-morbidities, virological, and serological tests, etc.). 

**Figure 7:**
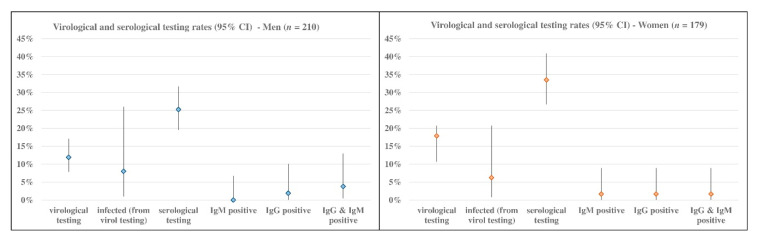
Dress-COV: virological and serological testing results.

**Figure 8:**
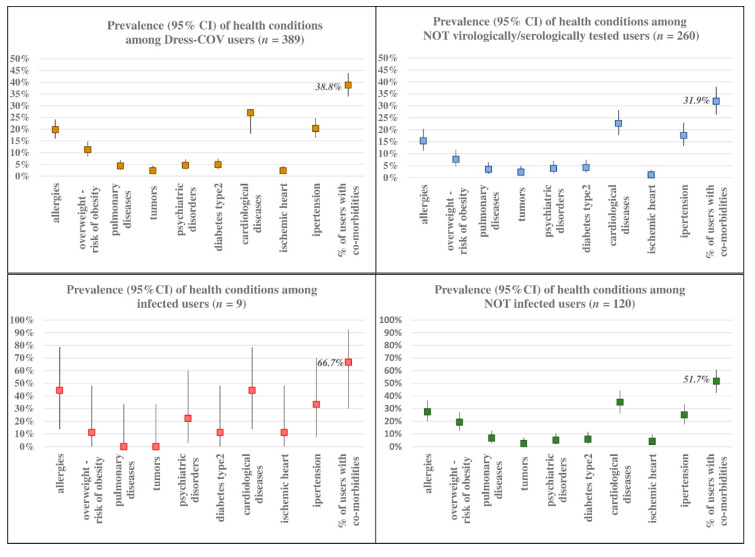
Dress-COV: co-morbidity rates.

By way of illustration, some data relating to the 27 August are reported:

(a) The prevalence of the Dress-COV users who have been virologically or serologically tested was 32.6% (*n* = 129, median age between 45 and 49 years) and the infected amounted to 7.0% (95% CI: 3.3–12.8%) corresponding to 9 subjects (median age between 55 and 59 years), with a higher prevalence of men (8.0% vs. 6.3%), as shown in Figure 7. Of those tested, 116 subjects were asymptomatic (89.9%; 95% CI: 83.3–94.4%) and, among the infected, 6 subjects were asymptomatic (66.7%; 95% CI: 29.9–92.5%). The most frequent symptoms are tiredness (*n* = 7), muscle soreness or aches, congestion or runny nose, diarrhea or nausea and headache (*n* = 6). Among the infected symptomatic subjects, tiredness and diarrhea or nausea are always reported. On average, excluding family members, 25.4% (95% CI: 17.7–34.4%) of the tested subjects reported to meet more than 30 persons during the day: this rate amounts to 37.5% (95% CI: 8.5–75.5%) among infected subjects.
(b) Co-morbidity rate among all of the users was 38.8% (95% CI: 33.9–43.4%): the subjects who tested positive for SARS-CoV-2 showed the highest rate of co-morbidity (66.7%; 95% CI: 29.9–92.5%). Cardiac diseases, psychiatric disorders, and type 2 diabetes are the main pathological categories that affected COVID-19 cases. As shown in Figure 8, the prevalence of subjects suffering from at least one allergy among positive users was 44.4% (95% CI: 13.7–78.8%).

Chronic allergic diseases are associated with tissue remodeling processes, and persistent inflammation may weaken the patient’s immune system, thus increasing susceptibility to infection [24]. 

A recent nationwide Korean cohort study [25] shows that the development of allergic respiratory diseases is associated with an increased risk of subsequent COVID-19, and/or worsening of clinical outcomes from COVID-19.

**Figure 9:**
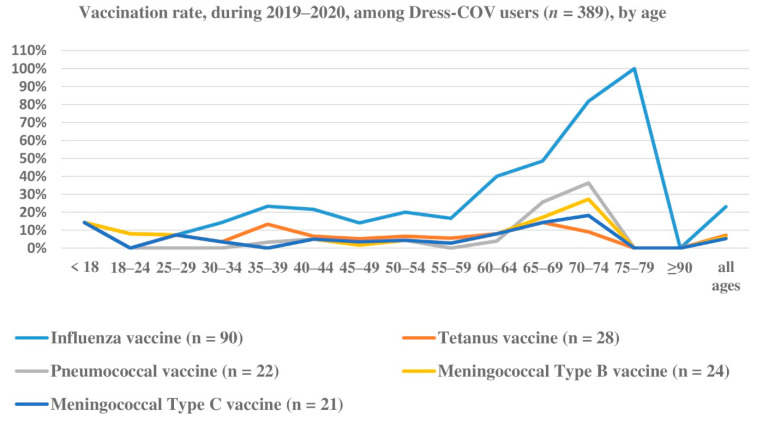
Dress-COV: vaccination rate (2019–2020) by age and type of vaccine.

(c) The vaccinations included in the survey are not mandatory in Italy [26], though the influenza vaccine is recommended for persons over 65 or who are chronically ill. Vaccination could be a proxy of self-healthcare, as well as a potential modifier of the risk of contagion. Vaccination rate (2019–2020) among all of the Dress-COV users differed by age and type of vaccination (Figure 9). The most frequent vaccination was the Influenza vaccine, followed by the tetanus vaccine. All rates were much lower than the vaccination coverage rates indicated by the Italian national health system, in the range of 75–95% for adults. This could be associated with the generalized vaccine hesitancy reported worldwide and worsened by the COVID-19 pandemic [27]. Vaccination coverage reported by the Italian Ministry of Health for the 2019–2020 flu season amounted to 54.5% [28].

**Figure 10:**
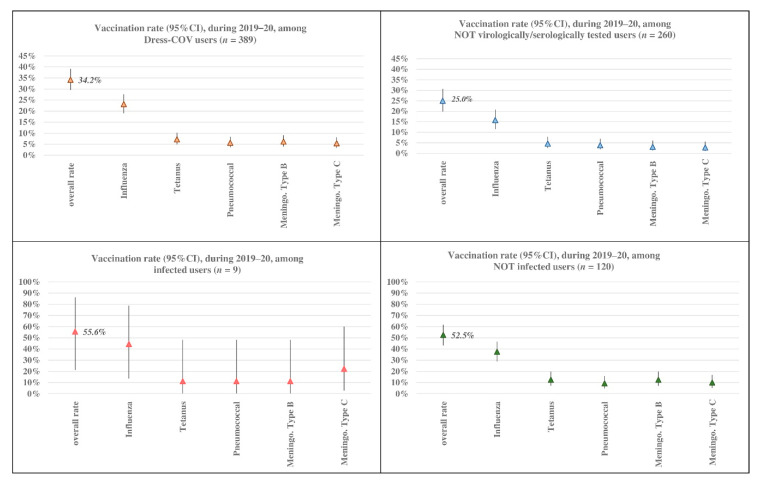
Dress-COV: vaccination rate (2019–2020) by propensity to tests and their results.

As shown in Figure 10, the overall vaccination rate was 34.2% (95% CI: 29.5–39.1%): only a quarter of not tested users had undergone vaccinations. Considering virologically or serologically tested users only, the vaccination rate was 52.7% (95% CI: 43.7–61.6%). This suggests that the propensity to vaccination could be associated with the propensity of doing virological or serological tests.

Co-morbidity rate significantly differs among vaccinated and not vaccinated users (Table 1), although the most relevant difference concerns users who did not undergo virological or serological tests. The vaccination rate amounted to 55.6% (95% CI: 21.2–86.3%) among infected subjects (*n* = 5) and, as shown in Table 1, this rate is not very different from that among tested negative subjects (52.5%; 95% CI: 43.2–61.7%). Infected subjects were also characterized by a more notable morbidity rate (80%; 95% CI: 28.4–99.5%). 

The limited number of infected subjects does not allow for evaluation of the relationship between vaccination and SARS-CoV-2 infection. These results could suggest a possible association between chronic conditions and both the vaccination propensity and the contagion risk.

(d) Most cases of aggressive influenza (i.e., body temperature higher than 39 degrees, muscle aches, congestion, persistent cough but without diagnosis of infection of the lungs) never progress to pneumonia (as the diagnosed complication of the aggressive flu), but those that do tend to be more severe. Thus, with the aim to collect all of the previous events of acute respiratory disease, we investigated both. The prevalence rates of previous influenza characterized by particularly aggressive symptoms and pneumonia were, respectively, 8.5% (95% CI: 5.9–11.7%) and 2.1% (95% CI: 0.9–4.0%) considering the total of the Dress-COV users. Those subjects with co-morbidities exhibit slightly higher rates, although are not statistically significant. In particular, the prevalence of previous events of aggressive influenza among COVID-19 infected users amounted to 50% (95% CI: 11.8–88.2%), higher than the rate concerning infected users without co-morbidities but not statistically significant (Table 2).
(e) Regardless of the statistical significance in the estimated rates, we aim to show the potential of the Dress-COV survey for profiling users referring to psychological aspects, propensity to cooperatively share information, and their use of main sources for public information (Table 3). The subjects who have undergone virological or serological testing reported higher ability for adaptation to context changes and higher propensity to share information. Independently from their testing status, approximately one-third of users reported the desire for more information by the experts, but some differences exist concerning their interest in general knowledge and the use of the different sources. Tested users, likely in accordance with their level of concern about the pandemic, show a greater use of all sources of information, with particular confidence in scientific sources.

It is critical to emphasize that current results are reported by way of illustration of the Dress-COV potential to promote citizens empowerment and informing public policy.

The analyzed dataset supporting the presented results is provided in Supplementary Materials.

## 4. Discussion 

Modern medicine identifies the key for successful healthcare as the “patient-centered” approach based on predictive, preventive, personalized, and participatory aspects [29]. The goal of personalized medicine can broadly be defined as the aim to account for those factors that make health and disease specific for each individual, but P4 medicine poses more emphasis on utilizing the “in silico” computational integration of big-data and “in socio” via patient participation in data collection [30]. Moreover, P4 medicine also focuses on healthy people being involved in a more expansive and life-long detection of early disease and risk factors, expressed by “billions of data points”, a staggering data dimensionality that should be modeled using simple hypotheses to identify patient-specific phenotypes [31].

This implies that not only the physician, but also the patients evolve their roles, shifting from a reactive position to a proactive approach to health and that this process should be supported by the massive sharing of information between patients and healthcare providers. It is especially helpful in managing the COVID-19 pandemic, where the real number of the pauci/asymptomatic infected people remains unclear and citizens’ participation could be critical. Moreover, communication during epidemics and pandemics is a key factor. Contradictory information and overexposure to sensationalist headlines from mass media and social media can affect people’s mental health and increase the general level of fear, leading to a global uncertainty on the most relevant and scientifically validated information about COVID-19, its symptoms and the most appropriate measures to protect one’s own health [32].

Conversely, strategies for health education and collaborative information pathways that focus on big communities could be efficient solutions for complementing syndromic surveillance and informal contact tracing.

This is particularly relevant in the current phase of the pandemic, as the number of cases and their contacts are underestimated, national contact tracing services are facing delayed notification of cases and quarantines. The spread of COVID-19 is growing, and the self-management of individual behaviors, in case of infection or un-notified quarantine, could be critical for containment of the risk of SARS-CoV-2 infection.

The Dress-COV has been designed and developed to help people reduce the uncertainty about an unknown disease and enable health providers to collect crucial data for better understanding the COVID-19 diffusion and characteristics. It is based on a widely distributed and embedded tool, i.e., Telegram. Moreover, the Dress-COV system aims to be a tool as a basis for a long-lasting collaboration with users. 

### 4.1. Principal Results

Compliance to the tool is the first result of our work. The exchange of personalized feedback, in-depth links, information about the evolution of the contagions interspersed with the administration of the daily questions, creates expectations on the users, motivating them to provide the subsequent answers in order to understand their risk profile and manage it. 

Risk estimation for becoming COVID-19 infected, currently based on the algorithm previously developed by Menni and colleagues [23], is a further inducement for the citizen’s continuing use and empowerment. The planned risk estimation upgrade towards ML algorithms based on users’ responses, improves the Dress-COV potential to support personalized prevention.

Furthermore, the Dress-COV system ensures a balance between gathering data from people for identifying characteristics of infected individuals while protecting their privacy, by using a series of key IDs managed within the National Health Care System (NHCS) only. According to the General Data Protection Regulation, in Italy, the management of sensitive personal data is legally authorized for organizations belonging to the NHCS only. The use of personal data for scientific purposes is permitted in anonymous form only, after the positive evaluation of an ethics committee. The high compliance with the tool shows the citizens’ willingness to participate in their own health management without any privacy concerns. This result is particularly relevant where, in Italy, data security of the Immuni app is still widely debated, as indicated by the relatively low number of downloads, notwithstanding the dissemination campaign promoted by the Italian government since May 2020. 

The second relevant outcome of the Dress-COV is its versatility. As it is happening in the rest of Europe, in Italy we are adapting to the phase of coexistence with the virus, the so-called phase 2, and it is fundamental to have systems that can adapt quickly to new needs and allow people to effect self-control of their individual risk, for directing their behaviors towards more protective habits. Dress-COV is a dynamic system and we are updating it, to manage the change in restrictions for social distancing related to the different trends of infection in geographical areas, and to increase the knowledge of COVID-19’s evolution. Currently, we are performing the second upgrade of the Dress-COV survey questions, in accordance with the most recent evidence regarding COVID-19 symptoms, long-term outcomes, type of swab performed, and concerning issues critical for managing the pandemic, i.e., swab/serological tests availability, booking timing, and notification time for test results. 

Another concrete result of our work concerns its potential towards territorial health providers. General Practitioners joined the Dress-COV system, considering it as an innovative tool for supporting their daily work in this pandemic, but also during their routine practice, and regional health providers are considering to integrate the Dress-COV with the health information systems already in use for many years, or with those specifically implemented for follow-up to the pandemic. In our opinion, the interest directed towards Dress-COV depends on the ability to easily share information between citizens and health services, as well as on the possibilities to customize potential reuse in different health scenarios. 

Focusing on the informative aspect of the Dress-COV, independent of the current statistical relevance of our results due to the limited number of registered users, we believe that its potential for collecting and sharing information on the dynamic process of COVID-19 (virological and serological testing, infected characteristics and risk factors) is valuable for the health system and for the citizen’s empowerment.

Dress-COV allows the estimation of the percentage of users undergoing virological and serological testing, the rate of infected and their profiles in terms of demographic features, symptomatology, co-morbidity, and other risk or protective factors (social contacts, vaccination, drug use, life habits, protective equipment use, etc.), independent from the notifications provided by the national surveillance system. In Italy, the Immuni app is driven by DoH notifications, and so it suffers from the delay of notification. 

By way of illustration only, our current statistical results indicate that infected subjects are mainly men (prevalence 8% vs. 6.3%) between 55 and 59 years of age, and most are asymptomatic (66.7%). The users who have undergone virological or serological tests showed symptoms, such as tiredness, muscle soreness or aches, congestion or runny nose, diarrhea or nausea, and headache: among the infected symptomatic subjects, tiredness and diarrhea or nausea are always reported.

The infected users also show the highest rate of co-morbidity, in particular exhibiting cardiac diseases, psychiatric disorders and type 2 diabetes, and their clinical complexity could impact the association between the probability of contagion and other risk/protective factors. Dress-COV also enables profiling users referring to aspects other than clinical ones. A quarter of tested subjects reported interacting with more than 30 people during the day and, considering the subset of the positive ones, this rate amounts to 37.5%.

Co-morbidity, vaccinations, acute events of aggressive flu or diagnosed pneumonia, psychological aspects and social habits are all considered among probable risk/protective factors that could be included in the Dress-COV aspects used for profiling healthy and infected citizens. All of these results must be validated, as the number of users increase and the ML methods can be applied. Information about the profiling results will be shared with users to address false beliefs and contradictory information from mass media and social media. 

Another result concerns the potential of the Dress-COV for profiling users relating to psychological factors, propensity to cooperatively share information and the use of the main sources of public information. These aspects are strategic when an individual citizen is intentionally and explicitly targeted as the agent of intervention for health promotion [33], considering individual susceptibility to disease risk, and the need for scientifically validated knowledge.

Furthermore, sharing of information between patients and healthcare providers is just as important for the COVID-19 pandemic as in facing those health priorities relating to chronic diseases and multi-morbidity, whose management is going to be even more challenging in the next months because of the long-term outcomes of SARS-CoV-2, and the delay in managing clinical and diagnostic plans concerning conditions other than COVID-19. For many decades, we have witnessed the rise in healthcare costs for long-term conditions or chronic diseases, added to the increasing shortages in the healthcare workforce [34], in which pandemic and isolation have become even more relevant issues. This is speeding up the shifting of the burden of primary healthcare to the family caregivers, without a concrete ability to capture patients’ perspectives of their health, and considering that patients’ priorities could be at variance with those of their healthcare providers [35].

The Dress-COV system, by integration, using an innovative approach of administering surveys and the use of specific functions of interaction with the users, or between the users and their GPs, aims to (a) increase the citizen’s consciousness and perception of wellbeing and of his own health status in order to promote empowerment; (b) emphasize the real social and care needs of citizens; (c) disseminate the most scientifically validated evidence for maintaining or increasing health; (d) support GPs in collecting alerts from their patients and quickly interact with them, and setting up preventive strategies towards patients who are at higher risk of contagion.

This supports the dissemination of the Dress-COV at the community level as an efficient strategy for promoting self-management of individual behaviors in many health fields. Existing funds and planning are in place to create a new version of the Dress-COV system that will be developed to investigate the association between dietary inflammatory levels and the risk of breast cancer, using a collaborative approach involving experts and female users. 

### 4.2. Limitation

The main limitation of the Dress-COV is the current number of users. Until now, the tool has only been locally deployed through the main social channels or informal communication networks. Moreover, Dress-COV has been promoted in the same period in which many other surveys have been administered to the general population for different purposes and other digital solutions, sponsored by industrial organizations which promoted large-scale information campaigns. The current limited number of users has delayed the risk assessment process development and the application of the ML functions. For this reason, we are performing the training of ML algorithms, by including additional data sources, coming from other national surveys [21,22] that investigate the same dimensions of Dress-COV. The output of the ML algorithms application will be the topic for further work providing additional validation of our initial results and their improvement.

A further limitation is one that the system shares with other mobile-based apps and digital platforms supporting surveillance activities for COVID-19 control. It concerns the dissemination among some age groups, such as older adults and children, who are less likely to have a mobile phone or have limited access to these applications. At this aim, we are designing a solution, aimed at caregivers, to allow multi-user mediated access to the Dress-COV system.

## 5. Conclusions

It is a common opinion that risk communication promotes community engagement, decreases uncertainty, and supports public health. As in every health field, communication in epidemics should reach the target population quickly, through the most accessible technologies. Compared with other digital solutions, Dress-COV is not a solution focused on tracing user contacts. It is mainly oriented toward promoting the knowledge and communication of COVID-19 risk, for improving the citizen’s self-empowerment and self-management of individual behavior, mainly in case of notification delay by the centralized services for COVID-19 surveillance. We believe that the current limitations of the Dress-COV system should be overcome to provide a solution for supporting a rapid and comprehensive sharing of information between the citizen and his GP, as an opportunity for promoting participatory disease surveillance, also potentially related to chronic diseases. 

## Figures and Tables

**Table 1:** Dress-COV: prevalence of morbidity among vaccinated and not vaccinated users.

	Prevalence of (Co)Morbidity among Vaccinated Subjects (95% CI)	Prevalence of (Co)Morbidity among Not Vaccinated Subjects (95% CI)
Not virological/serological tested	58.5% (45.6–70.6%)	22.7% (17.0–29.2%)
Virological/serological tested	52.9% (40.4–65.2%)	52.5% (39.3–65.4%)
Tested positive	80.0% (28.4–99.5%)	50.0% (6.8–93.2%)
Tested negative	50.8% (37.9–63.6%)	52.6% (39.0–66.0%)
Overall Dress-COVusers	55.6% (46.8–64.2%)	29.8% (24.3–35.8%)

**Table 2:** Dress-COV: Prevalence (95% CI) of previous events of pneumonia and flu in the past 12 months.

	Prevalence of Aggressive Flu in the Past 12 Months (95% CI)		Prevalence of Pneumonia in the Past 12 Months (95% CI)	
	With (co)morbidities (*n* = 14)	Without (co)morbidities (*n* = 19)	With (co)morbidities (*n* = 4)	Without (co)morbidities (*n* = 4)
Not virological/serological tested	10.8% (5.1–19.6%)	6.8% (3.6–11.5%)	3.6% (0.8–10.2%)	11% (0.1–4.0%)
Virological/serological tested	7.4% (2.4–16.3%)	11.5% (4.7–22.2%)	1.5% (0.0–7.9%)	3.3% (0.4–11.3%)
Tested positive	50.0% (11.8–88.2%)	33.3% (0.8–90.6%)	0.0% (0.0–45.9%)	33.3% (0.8–90.6%)
Tested negative	3.2% (0.4–11.2%)	10.3% (3.9–21.2%)	1.6% (0.0–8.7%)	1.7% (0.0–9.2%)
Overall Dress-COV users	9.3% (5.2–15.1%)	8.0% (4.9–12.2%)	2.6% (0.7–6.6%)	1.7% (0.5–4.2%)

**Table 3:** Dress-COV: frequency (95% CI) of some psychological aspects and use of informative sources between tested and not tested users.

	Not Virologically/Serologically Tested Users	All Virologically/Serologically Tested Users
Rate of users reporting high ability to adapt to context changes	57.3% (51.0–63.3%)	79.1% (71.0–85.7%)
Moods to the health emergency		
Uncontrollable anxious	7.7% (4.8–11.6%)	14.0% (7.8–20.7%)
Trust in provided information about risk reduction	60.0% (53.8–66.0%)	68.2% (58.3–75.8%)
Little concern	18.5% (13.9–23.7%)	15.5% (10.5–24.6%)
Bewilderment	6.9% (4.2–10.7%)	2.3% (0.5–7.1%)
Propensity to share information to solve the health emergency	55.8% (49.5–61.9%)	76.0% (67.6–83.1%)
Need of more information about pandemic by the experts	34.6% (28.8–40.7%)	37.2% (28.9–46.2%)
Use of information sources about the health emergency		
TV broadcast	35.8% (29.9–41.9%)	40.3% (31.8–49.3%)
Social network	57.3% (51.0–63.4%)	71.3% (62.7–78.9%)
Magazines and newspapers	25.8% (20.6–31.5%)	35.7% (27.4–44.6%)
TV news	48.8% (42.6–55.1%)	69.0% (60.2–76.8%)
Scientific sources	42.3% (36.2–48.6%)	74.4% (66.0–81.7%)

## Supplementary Materials

The following are available online at https://www.mdpi.com/1660-4601/17/23/8786/s1, Supplementary Materials A: Dress-COV Survey, Supplementary Materials B: Dataset Dress-COV Analysis.

## References

[1] WHO (2020). Novel Coronavirus (2019-nCoV) Situation Reports. https://www.who.int/docs/default-source/coronaviruse/situation-reports/20200831-weekly-epi-update-3.pdf?sfvrsn=d7032a2a_4.

[2] Puci M.V., Loi F., Ferraro O.E., Cappai S., Rolesu S., Montomoli C. (2020). COVID-19 Trend Estimation in the Elderly Italian Region of Sardinia. Front. Public Health.

[3] Ajelli M., Zhang Q., Sun K., Merler S., Fumanelli L., Chowell G., Simonsen L., Viboud C., Vespignani A. (2018). The RAPIDD Ebola forecasting challenge: Model description and synthetic data generation. Epidemics.

[4] Koetter P., Pelton M., Gonzalo J., Du P., Exten C., Bogale K., Buzzelli L., Connolly M., Edel K., Hoffman A. (2020). Implementation and Process of a COVID-19 Contact Tracing Initiative: Leveraging Health Professional Students to Extend the Workforce during a Pandemic. Am. J. Infect. Control..

[5] Dhillon R., Srikrishna D. (2018). When is contact tracing not enough to stop an outbreak?. Lancet Infect. Dis..

[6] Garg S., Bhatnagar N., Gangadharan N. (2020). A Case for Participatory Disease Surveillance of the COVID-19 Pandemic in India. JMIR Public Health Surveill..

[7] Guerrisi C., Turbelin C., Blanchon T., Hanslik T., Bonmarin I., Levy-Bruhl D., Perrotta D., Paolotti D., Smallenburg R., Koppeschaar C. (2016). Participatory Syndromic Surveillance of Influenza in Europe. J. Infect. Dis..

[8] Leal-Neto O., Santos F., Lee J., Albuquerque J., Souza W. (2020). Prioritizing COVID-19 tests based on participatory surveillance and spatial scanning. Int. J. Med. Inform..

[9] Frydman G. A Patient-Centric Definition of Participatory Medicine. https://participatorymedicine.org/epatients/2010/04/a-patient-centric-definition-of-participatory-medicine.html.

[10] Ferreira M.S., Castiel L.D. (2009). Which empowerment, which Health Promotion? Conceptual convergences and divergences in preventive health practices. Cad Saúde Pública.

[11] Kumar S., Preetha G. (2012). Health promotion: An effective tool for global health. Indian J. Community Med..

[12] Eysenbach G. (2003). SARS and Population Health Technology. J. Med. Internet Res..

[13] Prainsack B. (2013). Let’s get real about virtual: online health is here to stay. Genet. Res..

[14] Auffray C., Charron D., Hood L. (2010). Predictive, preventive, personalized and participatory medicine: Back to the future. Genome Med..

[15] Agusti A., Sobradillo P., Celli B.R. (2011). Addressing the Complexity of Chronic Obstructive Pulmonary Disease. Am. J. Respir. Crit. Care Med..

[16] Data Learn Lab 2020 Progetto DRESS. https://www.datalearnlab.it/news/2020/03/26/Progetto-DRESS.html.

[17] Franchini M., Pieroni S., Cutilli A., Caiolfa M., Naldoni S., Molinaro S. (2019). The Individual Profile of Pathology as a New Model for Filling Knowledge Gaps in Health Policies for Chronicity. Front. Med..

[18] Prainsack B. (2014). The Powers of Participatory Medicine. PLoS Biol..

[19] Cooper A., Reimann R., Cronin D., Noessel C. (2014). About Face: The Essentials of Interaction Design.

[20] Breiman L. (2001). Random Forests. Mach. Learn..

[21] Adorni F., Prinelli F., Bianchi F., Giacomelli A., Pagani G., Bernacchia D., Rusconi S., Maggi S., Trevisan C., Noale M. (2020). Self-reported symptoms of SARS-CoV-2 infection in a non-hospitalized population: Results from the large Italian web-based EPICOVID19 cross-sectional survey. (Preprint). JMIR Public Health Surveill..

[22] Bastiani L., Fortunato L., Pieroni S., Bianchi F., Adorni F., Prinelli F., Giacomelli A., Pagani G., Maggi S., Trevisan C. (2020). EPICOVID19: Psychometric assessment and validation of a short diagnostic scale for a rapid Covid-19 screening based on reported symptoms. J. Med. Int. Res..

[23] Menni C., Valdes A.M., Freidin M.B., Sudre C.H., Nguyen L.H., Drew D.A., Ganesh S., Varsavsky T., Cardoso M.J., Moustafa J.S.E.-S. (2020). Real-time tracking of self-reported symptoms to predict potential COVID-19. Nat. Med..

[24] Galli S.J., Tsai M., Piliponsky A.M. (2008). The development of allergic inflammation. Nat. Cell Biol..

[25] Yang J.M., Koh H.Y., Moon S.Y., Yoo I.K., Ha E.K., You S., Kim S.Y., Yon D.K., Lee S.W. (2020). Allergic disorders and susceptibility to and severity of COVID-19: A nationwide cohort study. J. Allergy Clin. Immunol..

[26] Il Calendario Vaccinale del Piano Nazionale di Prevenzione Vaccinale 2017–2019. http://www.salute.gov.it/imgs/C_17_pagineAree_4829_listaFile_itemName_0_file.pdf.

[27] Odone A., Bucci D., Croci R., Riccò M., Affanni P., Signorelli C. (2020). Vaccine hesitancy in COVID-19 times. An update from Italy before flu season starts. Acta Biomed. Atenei Parm..

[28] Dati Coperture Vaccinali. http://www.salute.gov.it/portale/influenza/dettaglioContenutiInfluenza.jsp?lingua=italiano&id=679&area=influenza&menu=vuoto.

[29] Lejbkowicz I., Caspi O., Miller A. (2012). Participatory medicine and patient empowerment towards personalized healthcare in multiple sclerosis. Expert Rev. Neurother..

[30] Green S., Vogt H. (2016). Personalizing medicine: Disease prevention in silico and in socio. Hum. Mente.

[31] Bousquet P.J., Anto J.M., Sterk P.J., Adcock I.M., Chung K.F., Roca J., Agusti A., Brightling C., Cambon-Thomsen A., Cesario A. (2011). Systems medicine and integrated care to combat chronic noncommunicable diseases. Genome Med..

[32] Fagherazzi G., Goetzinger C., Rashid M.A., Aguayo G.A., Huiart L. (2020). Digital Health Strategies to Fight COVID-19 Worldwide: Challenges, Recommendations, and a Call for Papers. J. Med. Internet Res..

[33] Simmons L.A., Dinan M.A., Robinson T.J., Snyderman R. (2012). Personalized medicine is more than genomic medicine: confusion over terminology impedes progress towards personalized healthcare. Pers. Med..

[34] Gibbons M.C., Shaikh Y., Weaver C., Ball M., Kim G., Kiel J. (2016). The Patient of the Future: Participatory Medicine and Enabling Technologies. Healthcare Information Management Systems.

[35] Upshur R.E., Tracy S. (2008). Chronicity and complexity: Is what's good for the diseases always good for the patients?. Can. Fam. Physician.

