# Supplementary material for: Shifting the Paradigm: The Dress-COV Telegram Bot as a Tool for Participatory Medicine
Source: Int J Environ Res Public Health. 2020 Nov 26;17(23):8786. doi: 10.3390/ijerph17238786 (PMC7729623; doi:10.3390/ijerph17238786)
Supplement: Supplementary file 1 [file ijerph-17-08786-s001.zip › Supplementary A_Dress-COV Survey.docx]

**Dress-COV Survey**

The Dress-COV survey consists of 226 questions investigating sources of exposure that could lead to the contagion by the SARS-COV-2 and the personal aspects that could promote the onset of severe complications of the Covid-19 syndrome. Moreover, Dress-COV aims to evaluate the diagnostic pathway (virological and serological testing, quarantine) followed by the user and the possible occurrence of hospitalization for Covid-19 complications. Some questions also concern those Covid-19 symptoms currently known. The questions are grouped in about 30 clusters of 7 items administered day by day. The questions are grouped by survey sections but the order in which they are presented is conditioned by (1) the responses provided by the user, (2) the period of COVID-19 emergency. All the questions are close-ended and users can select pre-defined responses, such as “Male/Female” or among a set of multiple choice; users are never required to write text. The questions are clear and simple and the respondents don’t need to spend much time on reading.

Some control questions are submitted to assess the level of reliability of the answers.

The following session provides the DRESS-COV survey questions.

The symbol ***#***, when present, indicates that the same type of data is collected also by the external EPICOVID19 dataset, composed of several variables with the same meaning of the Dress-COV ones.

# Socio-demographic ***#***

- How old are you? *Response options: lower than 18;19-24;25-29; etc.*
- What is your gender? *Response options: male; female*
- What is your educational qualification? *Response options:* *none; primary; secondary; etc.*

# Occupational exposure

- Are you a student? ***#***
- Are you a retiree? ***#***
- In which of the following professional fields does your last job position fit? *Response options: Intellectual and scientific professions; Healthcare professions; etc.;* ***#***
- Are you unemployed? ***#***
- Are you a housewife?
- Are you employed? ***#***
- Do you work in a hospital as a healthcare professional? *Response options: doctor, nurse, biologist, etc.;*
- Do you work in a hospital as a NON-healthcare staff? *Response options: administrative, porter, cleaner, etc.*
- Do you work in a large shopping mall?
- Do you work in a small shop?
- Do you work in one of these sectors or structures? *Response options: Residential house for the elderly; day care center for the elderly / Alzheimer's, etc.*
- Are you a General Practitioner?
- Are you a Family Pediatrician?
- Do you need to leave the home for work needs? ***#***

Indicate how often you leave home for work. *Response options: once a week, 2 to 3 times a week, 4 to 6 times a week, once a day, more than once a day, never*

- Are you equipped with professional ffp2 masks?
- Are you equipped with disposable gloves?
- Are you equipped with a non-woven suit?
- Are you equipped with gowns?

# Family and housing

- Your family (including you) is made up of: choose whether 2,3,4, 5 or more people***#***
- Except you, are there people over 75 in your household?
- Except you, are there people under the age of 14 in your household?
- Except you, are there people in your family who work in hospitals?
- Except you, are there people in your household who work in shopping malls, shops?
- Do you live in an Assisted Healthcare Residence?
- Do you live in an isolated house? ***#***
- Indicate if you are self-reliant or someone is taking care of your basic needs. ***#*** *Response options:1,2*
- When you got sick, were you able to go to your family doctor in a short time?
- When you got sick, were you able to contact relatives - neighbours in a short time?
- When you got sick, were you able to go to other people in a short time?
- During this period, would you like to receive shopping at home?
- During this period would you like to receive pharmaceuticals at home?
- During this period would you like to have someone helping you with house cleaning?
- During this period would you like to have someone accompanying you to medical examinations or cycles of therapy?
- During this period would you like to have someone checking your health daily, even by phone call?
- During this period would you like someone staying with you?
- During this period would you like to ask an expert questions about the current situation?

# Environmental exposure

- Have you shared the geographical location where you normally spend most of the day? ***#***
- Is the geographic location you shared in an area with tourist influx? *Response options: poor, medium, high, I don't know****#***
- Is the geographic location you shared in a road traffic area? *Response options: poor, medium, high, I don't know****#***
- Do you live or work in environments with temperatures above 18 degrees?
- Do you live or work in humid environments?

# Life and social habits

- Are you currently working from home? ***#***
- Do you go out to take the dog outside?
- Do you go out for needs certified by your doctor?
- Do you go out for other family needs?
- Do you leave home for other reasons?
- Excluding going out for work and shopping reasons, indicate how often you leave the house. *Response options: once a week, from 2 to 3, etc.*
- When you go out, how often do you use public transport? *Response options: once a week, from 2 to 3, etc.* ***#***
- When you go out how often do you use private means of transport (car, bicycle)? *Response options: once a week, from 2 to 3, etc.*
- Have you to leave home to assist non self-sufficient people?
- How often do you leave home to assist other people? *Response options: once a week, from 2 to 3, etc.*
- On average, excluding family members, how many people do you meet during the day? *Response options: lower than 10, from 11 to 20, etc.*
- During this period, are you the one who does the shopping within your family?
- Where do you mainly go for your shopping?
- On average, how long do you stay in queue before shopping?
- On average, how many shops do you visit every time you do shopping?
- Indicate how often you go out for shopping
- When you go out, do you wear a mask made by you?
- When you go out, do you wear a surgical mask?
- When you go out, do you wear a professional mask?
- When you go out, do you wear gloves?
- Do you currently feed yourself properly with respect to the energy you spend on a daily basis?
- Do you drink at least 1.5 liters of water a day?
- Do you currently make physical exercise at home?
- Do you use magazines and newspapers to know about the most effective strategies for keeping yourself healthy?
- Do you use TV news to know about the most effective strategies for keeping yourself healthy?
- Do you use TV broadcast to know about the most effective strategies for keeping yourself healthy?
- Do you use social network to know about the most effective strategies for keeping yourself healthy?
- Do you use scientific channels to know about the most effective strategies for keeping yourself healthy?
- Do you use magazines and newspapers to know about the current health emergency?
- Do you use TV news to know about the current health emergency?
- Do you use TV broadcast to know about the current health emergency?
- Do you use social network to know about the current health emergency?
- Do you use scientific channels to know about the current health emergency?
- Before the lockdown, did you follow a healthy, varied, balanced diet, rich in fruit and vegetables? *A healthy diet means a diet low in fat, with a limited consumption of salt and sausages and which includes at least 5 portions a day of fruit and vegetables*
- Before the lockdown, did you practice physical activity*?* ***#*** *Physical activity includes structured physical exercise (e.g. gym) and other activities that involve body movement and are carried out in the context of play, work, active transport (traveling by bicycle or on foot), householding and recreational activities.*
- Do you practice sport at competitive level? by sporting activity we mean planned physical exercise performed regularly
- Do you practice sports at an amateur level? (including fitness)
- Does your sport involve the use of equipment (including the ball)?
- Have you started practicing your usual sporting activity again?
- *After the end of lockdown:*
  - Where do you usually train?
  - How often do you train?
  - During your sport activity do you respect the social distancing rule (1.8m)?
  - Is it mandatory to disinfect your hands before training?
  - Is it mandatory to undergo temperature control before training?
  - Is it mandatory to submit a symptom-free certification before training?
  - Do you have to certify your presence in any way before training?
  - Think about your typical training before the health emergency and compare it with the current one: do they have the same characteristics in terms of physical and technical exercises?
  - Can you currently use the changing rooms to change your clothes before training?
  - Can you currently use the shower after training?

# Anamnesis and comorbidities

*For all questions in this section the response options are Yes;No;I don’t know*

- In the past 12 months have you had pneumonia? ***#***
- In the past 12 months have you had any particularly aggressive forms of flu? ***#***
- In 2019-20 did you undergo any vaccinations? ***#***
- In 2019-20 did you get the tetravalent vaccine - anti-measles, anti-mumps, anti-rubella and anti-varicella?
- In 2019-20 did you get the pneumococcal vaccine? ***#***
- In 2019-20 did you get the meningococcal B vaccine?
- In 2019-20 did you get a meningococcal C vaccination?
- In 2019-20 did you get the rotavirus vaccine?
- In 2019-20 did you get the flu vaccine? ***#***
- In 2019-20 did you get a single tetanus vaccine?
- In the past 5 years have you suffered from hypertension? ***#***
- In the past 5 years have you suffered from atrial fibrillation?
- In the past 5 years have you suffered from ischemic heart disease?
- In the past 5 years have you suffered from heart failure?
- In the past 5 years have you suffered from stroke?
- In the past 5 years have you suffered from COPD (chronic obstructive pulmonary disease)? ***#***
- In the past 5 years have you suffered from type 2 diabetes? ***#***
- In the past 5 years have you suffered from dementia? ***#***
- In the past 5 years have you suffered from chronic liver disease? ***#***
- In the past 5 years have you suffered from kidney failure? ***#***
- In the past 5 years have you suffered from neoplasms (tumors)? ***#***
- In the past 5 years have you had psychiatric problems? ***#***
- In the last 5 years have you been addicted to psychotropic substances?
- In the past 5 years were you waiting for a transplant or have you had one? ***#***
- In the past 5 years have you had acquired immunodeficiency syndrome (HIV-AIDS)?
- In the past 5 years have you had heart disease? ***#***
- In the past 5 years have you had chronic respiratory diseases? ***#***
- In the past 5 years have you had chronic gastroenterological diseases?
- In the past 5 years have you had any neurological diseases?
- In the past 5 years have you had autoimmune diseases? ***#***
- In the past 5 years have you had endo-metabolic diseases?
- In the past 5 years have you had rare diseases?
- Are you allergic? ***#***
- Are you severely overweight or at risk of obesity? ***#***
- Are you pregnant? ***#***
- Do you smoke traditional cigarettes, e-cigarettes, vaping and tobacco heaters? ***#***
- Have you ever been a smoker?
- Are you often exposed to secondhand smoke?

# Medicines

*For all questions in this section the response options are Yes;No;I don’t know*

- Do you regularly take food supplements? ***#***
- Do you regularly take sleep supplements or medications?
- Do you regularly take birth control pills or hormone replacement therapy? ***#***
- Do you regularly take any medications?
- Do you regularly take Sartani-type antihypertensive drugs?
- Do you regularly take ACE inhibitor type antihypertensive drugs?
- Do you regularly take antihypertensive drugs such as calcium inhibitors (e.g. Nifedipine, Diltiazem, JTV-519, Verapamil, Bepridil)?
- Do you regularly take blood pressure medications? ***#***
- Do you regularly take medications to treat cholesterol? ***#***
- Do you regularly take medications to treat diabetes? ***#***
- Do you regularly take anticancer drugs? ***#***
- Do you regularly take steroids?
- Do you regularly take thyroid medications? ***#***
- Do you regularly take anti-inflammatory drugs? ***#***
- Do you regularly take anti-anxiety drugs, sedatives? ***#***
- Do you regularly take antidepressant medications? ***#***
- Do you regularly take Aspirin? ***#***
- Are you taking immunosuppressive drugs?
- Are you undergoing chemotherapy cycles?
- Are you undergoing radiotherapy cycles?

# Symptoms

*For all questions in this section the response options are Yes;No;I don’t know*

- Have you had fever in the last few days? ***#***
- Have you been tired in the last few days?
- Have you had muscle aches and pains in the past few days? ***#***
- Have you had shortness of breath in the past few days? ***#***
- Have you had nasal congestion in the past few days? ***#***
- Have you had a runny nose in the past few days? ***#***
- Have you had sore throat in the past few days? ***#***
- Have you had diarrhea or nausea in the past few days? ***#***
- Have you had headache in the past few days? ***#***
- Have you had hemoptysis (blood from the respiratory tract) in the past few days?
- Have you had lack of smell in the past few days? ***#***
- Have you had taste deficit in the past few days? ***#***
- Have you had hypotension (low blood pressure) in the past few days?
- Have you had tachycardia in the past few days? ***#***
- Have you had any sleep disorders in the past few days?

# Serological testing

- Did you take the serological test? ***#*** *Response options: Yes, positive to IgM; Yes, positive to IgG; Yes, I don’t know the results; Yes, I don’t know if positive to IgM or IgG; No*
  - Are you a plasma donor?

# Virological testing

- Did you do the swab? ***#*** *Response options:* *Yes, positive; Yes, negative; Yes, I don’t know result; No*
- When you had the swab, did you have any symptoms?
- When you had the swab, did you have fever over 38 degrees?
- When you had the swab, were you experiencing unusual tiredness?
- When you had the swab, did you have muscle soreness or aches?
- When you had the swab, did you have shortness of breath?
- When you had the swab, did you have nasal congestion or runny nose?
- When you had the swab, did you have sore throat?
- When you had the swab, did you have diarrhoea or nausea?
- When you had the swab, did you have headache?
- When you had the swab, did you have haemoptysis (bleeding from the respiratory tract)?
- When you had the swab, did you have lack of smell?
- When you had the swab, did you have a taste deficit when you swab?
- When you had the swab, did you have hypotension (low blood pressure)?
- When you had the swab, did you have tachycardia?
- Did you have control swabs to establish your recovery?

# Quarantine

- Are you in quarantine?
- When you started quarantine, did you have any symptoms?
- When you started quarantine, did you have fever over 38 degrees?
- When you started quarantine, did you have unusual tiredness?
- When you started quarantine, did you have muscle soreness or aches?
- When you started quarantine, were you short of breath?
- When you started quarantine, did you have nasal congestion or a runny nose?
- When you started quarantine, did you have sore throat?
- When you started quarantine, did you have diarrhea or nausea?
- When you started quarantine, did you have headache?
- When you started quarantine, did you have hemoptysis (blood from the respiratory tract)?
- When you started quarantine, did you have lack of smell?
- When you started quarantine, did you have taste deficit?
- When you started quarantine, had you hypotension (low blood pressure)?
- When you started quarantine, did you have tachycardia?

# Hospitalisation

- Have you been hospitalized for suspected or confirmed Covid 19 infection? ***#***
- Have you been intubated or transferred to an ICU?
- How long was your hospitalization in total?
- Did you have a control swab prior to discharge?

# Psychological profile

- On a 1 to 10 scale, can you evaluate whether in life you achieved most of the goals set for yourself? When answering this question, choose the level that best describes you, considering that the value 10 indicates a full achievement.
- When you think about this health emergency. ***#*** *Response options: you are overwhelmed by anxiety / fear of contagion; you believe that the precautionary indications given to us can reduce our individual risk; you're not particularly worried; you don't know what to think*
- On a 1 to 10 scale, can you evaluate your ability to adapt to context changes?
- On a 1 to 10 scale, how much each of us can contribute to the resolution of the emergency using their skills or availabilities?
- On a 1 to 10 scale, providing the information requested in this survey is a way to help resolve the emergency?
- In your opinion, could this emergency provide some prospects for improvement of our future?
- During this period, at the idea of going out you feel: uncontrollable anxious, worried, not worried at all. *Response options: 1;2;3*
- Thinking back to the lockdown, at the idea of going out you feel: uncontrollable anxious, worried, not worried at all *This question was sent to users in alternative to the previous one, after lockdown end in Italy*
- Thinking back to the lockdown, did you spend your time reading and studying?
- Thinking back to the lockdown, did you spend your time watching TV?
- Thinking back to the lockdown, did you spend your time listening to music?
- Thinking back to the lockdown, did you spend time doing physical exercise at home?
- Thinking back to the lockdown, did you spend time talking with relatives and friends via telephone, community on the net?
- Thinking back to the lockdown, did you spend your time getting bored?
- Thinking back to the lockdown, did you spend your time cooking?
- Thinking back to the lockdown, did you spend your time embroidering or knitting?
- Thinking back to the lockdown, did you spend your time helping children or grandchildren with schoolwork?
- Thinking back to the lockdown, did you spend your time doing DIY?
- Thinking back to the lockdown, did you spend your time gardening?
- Thinking back to the lockdown, did you spend your time doing small maintenance work for your house?
- Thinking back to the lockdown, did you spend your time doing house holding?
- Thinking back to the lockdown, did you spend your time working from home?
- Thinking back to the lockdown, did you go out because you couldn't stay alone for all day?
